# Associated factors with patient-reported unmet food needs among emergency department adult patients – A social need perspective

**DOI:** 10.1016/j.pmedr.2022.101974

**Published:** 2022-09-07

**Authors:** Nasser Sharareh, Andrea S. Wallace, Ben J. Brintz, Neng Wan, Jia-Wen Guo, Bob Wong

**Affiliations:** a Department of Population Health Sciences, Spencer Fox Eccles School of Medicine at University of Utah, Salt Lake City, UT, USA; b College of Nursing, Spencer Fox Eccles School of Medicine at University of Utah, Salt Lake City, UT, USA; c Internal Medicine, Spencer Fox Eccles School of Medicine at University of Utah, Salt Lake City, UT, USA; d Geography Department, University of Utah, Salt Lake City, UT, USA

**Keywords:** Social needs screening, Social determinants of health, Food insecurity, Geographic information systems

## Abstract

Food insecurity is a complex problem affected by a number of factors from individual to societal. While individual-level demographic information and population-level social determinants of health (SDoH) are commonly used to identify patients at risk of food insecurity and to direct resources, a more comprehensive understanding of food insecurity requires integrating multi-level data. Our goal is to identify factors associated with food insecurity using patient, health system, and population level data. Between January 2019 and April 2020, we screened adult patients visiting an academic health sciences emergency department in Utah using a 10-item social needs screener. Patients’ demographic data were linked to their screener responses. ZIP Code-level food-related SDoH such as accessibility to food providers, measured by geographic information systems methods, were assigned to patients. We then applied multilevel logistic regression modeling to identify factors associated with unmet food needs at two different levels—individual and ZIP Code. Unmet food needs were identified by asking patients if they felt there was not enough money for food in the last month, which grossly represents food insecurity. On a sample of 2,290 patients, 21.61% reported unmet food needs. Patient-reported housing, medical care, and utility needs along with Supplemental Nutrition Assistance Program participation and primary care provider utilization were highly associated with unmet food needs. Our efforts to identify the population at risk of food insecurity should be centered around patient-reported social needs. Our results suggest that addressing food insecurity in health care settings should include assessing social needs in primary care.

## Highlights

• Food insecurity is a complex problem.
• Patient-reported food needs are intertwined with other social needs.
• Addressing food insecurity should be centered around patient-reported social needs.

## 1 Introduction

Food insecurity is the lack of access to adequate food for an active healthy life. (Coleman-Jensen et al., 2020) It impacted 10.5 % of the United States (U.S.) households at some time during 2020. (Coleman-Jensen et al., 2020) Food insecurity at the community level is a social determinant of health that is annually estimated by Feeding America using community-level characteristics (e.g., unemployment rate, disability rate). (America, 2020) At the household level, food insecurity is measured using an 18-item or a 10-item questionnaire administered by the United States Department of Agriculture (USDA) (Coleman-Jensen et al., 2020). Simpler methods such as Hunger Vital Sign (Hager et al., 2010) contain only two questions that can estimate USDA’s food insecurity measures with a sensitivity of 97 %. In addition, individual-level food insecurity, which is the focus of our paper, is commonly assessed during a healthcare visit by either using the Hunger Vital Sign (Escobar et al., 2021) or a 1-item questionnaire that identifies unmet food needs by asking about patients’ financial barriers to accessing food. (De Marchis et al., 2019).

Currently, in order to identify the risk factors associated with food insecurity at the individual level, we rely on demographic factors and utilization of federal nutrition assistance programs (Martinez et al., 2018, Chilton et al., 2009, Ettinger de Cuba et al., 2019) and commonly ignore the impact of other individual-level social needs on food insecurity. (Sharareh and Wallace, 2022) Social needs such as housing, transportation, and healthcare are intertwined with unmet food needs. A cocktail of social needs puts people in a closed loop of problems hard to address simultaneously. For instance, among households served by the Feeding America network, it is reported that 66 % of people must choose between food and medical care, 69 % between food and utilities, 67 % between food and transportation, and 57 % between food and housing. (Feeding America, 2014) Also in a sample of San Diego’s population, people with utility needs and housing issues (i.e., homeless-sheltered) were 29 % more likely to have food insecurity. (Yousefi-Rizi et al., 2021) In addition, social needs indicate real-time needs and gaps that affect one’s health, (Glasheen, 2019) however, patient-reported social needs are left out of our food insecurity estimations and discussions of intervention design. (Sharareh and Wallace, 2022) Moreover, less is known about the insight patient-reported social needs might provide along with individual-level demographics and population-level social determinants of health (SDoH). Therefore, we need to investigate this problem from a multi-level perspective.

Addressing individual-level food insecurity requires intervening at the community level to address SDoH that impact food insecurity, including access barriers. (Castrucci and Auerbach, 2019) For instance, the availability of food pantries in low-income areas without a supermarket could increase access to healthy food options (Mabli et al., 2013) and eventually improve food security; or many food-insecure households are ineligible to enroll in Supplemental Nutrition Assistance Program (SNAP) due to slightly higher income which increases food insecurity rates among those households. (Schanzenbach et al., 2016) Nevertheless, because social needs screening is being widely adopted in health systems, patient-reported unmet food needs at the individual level may be a useful tool for understanding the prevalence and associations of food insecurity to inform targeted interventions at the individual- and community- levels. (Alderwick and Gottlieb, 2019) This is a critical need as the U.S. healthcare system is also looking for cost-effective tools and approaches to identify social needs.

The benefits of social needs screening are well documented. For example, social needs screening in pediatric primary and urgent care clinics and subsequently providing information related to community and federal resources addressing needs to caregivers have decreased the number of social needs. (Gottlieb et al., 2016) Social needs screening in primary care providers (PCP) settings has also improved the communication between providers and patients (Tong et al., 2018) and will produce data that can be used to better manage chronic conditions. (Heller et al., 2021) In addition, patients have shown interest in social needs screening (Hsu et al., 2020, Rogers et al., 2020) and agree that social needs impact their health. (Rogers et al., 2020) However, screening for food insecurity in U.S. healthcare settings is limited (Fraze et al., 2019) and most of the rigorous studies had been done in pediatric settings. (De Marchis et al., 2019) Considering all these benefits, our efforts are still focused on identifying at-risk patients based on geography, (Morland et al., 2002, Smith et al., 2018) or demographic information. (Morris et al., 2016).

While a patient-reported unmet food need could be an indicator of individual-level food insecurity, less is known about how patient-reported social needs can provide new insights about food insecurity after controlling for critical factors at multi-levels. To the best of our knowledge, this is the first research paper that explores the impact of patient-reported social needs on unmet food needs in a clinical setting (in this case, an emergency department (ED)) after accounting for a rich source of covariates. The conceptual framework underlying this work is that interventions could be better tailored by incorporating data from multiple levels, and specifically, by considering real-time patient-reported social needs in our decision-making.

## 2 Study and data methods

This is a secondary data analysis leveraging data collected during universal social needs screening at an academic health sciences center ED in Utah between January 2019 and April 2020 using the 10-item, low literacy Screener for Intensifying Community Referrals for Health (SINCERE) on touchscreens provided in English. (Guo et al., 2021) The screener assesses ten patient-reported social needs in the last month (see Table 1) and uses a yes/no/prefer not to answer response format. The questions were developed through a series of meetings with different stakeholders and by following clear communication and low-literacy principles. (Wallace et al., 2020) The process used to develop SINCERE is described elsewhere. (Guo et al., 2021, Wallace et al., 2020) Although there are many screening tools to be adopted in clinical settings, the pragmatic and psychometric properties of such tools are unknown. (Henrikson et al., 2019) However, the SINCERE screener is a pragmatic and psychometrically sound tool. (Guo et al., 2021) We are also aware of the difference between social risk factors and social needs. (Alderwick and Gottlieb, 2019, Green and Zook, 2019) For instance, a screening tool can identify several social risk factors such as food and housing, but the patient may tell the provider that her most pressing issue is to address her mental health problems. Since these questions were designed to connect people with appropriate care, they are conceptualized as capturing social needs.

**Table 1:** SINCERE screener used to evaluate social needs in the last month.

Social Needs	Question
Transportation	1. Have you not seen a doctor because you didn't have a way to get to the clinic or hospital?
Medical Visit	2. Have you needed to see a doctor but could not because it costs too much?
Medication	3. Did you not take medications to save money?
Food	4. Did you feel there was not enough money for food?
Clothing/Furniture	5. Did you feel there was not enough money for items like clothing or furniture?
Utilities	6. Was there a time when you were not able to pay your utility bills?
Rent/Mortgage	7. Was there a time when you were not able to pay your mortgage or rent?
Housing	8. Have you slept outside, in a shelter, in a car, or any place not meant for sleeping?
Employment	9. Have you been unemployed and looking for work?
Childcare/Eldercare	10. Have problems getting child care or elder care made it difficult for you to work or get to appointments?

The Institutional Review Board at the University of Utah approved the study for the protection of human subjects concerning safety and privacy.

**Outcome variable**: A Patient-reported unmet food need is used as the binary outcome variable. Patients were asked if they felt there was not enough money for food in the last month (Guo et al., 2021, Wallace et al., 2020) (question 4 in Table 1), and they were assigned 1 if their answer was yes. This question closely represents the first question on the 2-item food insecurity screening tool, named Hunger Vital Sign, developed by Hager, Quigg, et al. (Hager et al., 2010): “Within the past 12 months we worried whether our food would run out before we got money to buy more.” This question alone can represent food insecurity by 92.5 % sensitivity. (Hager et al., 2010).

**Covariates**: We utilized other social needs (binary variables) along with Individual-level demographics (age, gender, race, and ethnicity) and health utilization factors (continuous variables for ED, PCP, and hospitalization utilization in the past 90 days), and ZIP Code level data (including accessibility to food providers, metro/nonmetro status, median household income, education level, SNAP utilization, and health insurance) as covariates (i.e., independent variables). Healthcare utilization was evaluated for the past 3 months to incorporate the patients’ behavior into the analysis. Food providers’ addresses were obtained from United Way of Salt Lake. (United Way. United Way of Salt Lake., 2022) Calculation of accessibility to food providers is explained in the next section. The metro/nonmetro status of the patient’s ZIP Code was identified by using the rural–urban commuting area codes. (USDA's Economic Research Service (ESR). Rural-Urban Commuting Area Codes, 2020) Other ZIP Code-level data were acquired from the census, using the 2019 5-year estimates. (Census, 2022) SNAP utilization indicates the rate of households receiving SNAP in the last year in each ZIP Code. Please see the appendix for detailed information about all the covariates.

### 2.1 Statistical and GIS analysis

We fitted a multilevel logistic regression model (Gelman and Hill, 2006) to estimate the odds of unmet food needs using covariates at both the individual and the ZIP Code level. We removed covariates that were highly correlated with other factors (i.e., more than 0.7; we removed the ones that were also moderately correlated with other factors) from analyses including clothing/furniture and rent/mortgage needs at the individual level, and median household income, education level, and health insurance at the ZIP Code level. We included a random ZIP Code intercept to account for within-ZIP Code correlation. To test our hypothesis, we added the covariates group by group, starting from individual-level demographic and health utilization factors, then ZIP Code-level data, and finally social needs.

In addition, we utilized Geographic Information System (GIS) methods and ArcGIS Pro 2.7.3 (ESRI, Redlands, CA) to measure the spatial accessibility of food providers for every ZIP Code in Utah. For these analyses, we required two inputs as demand and supply. For demand, we measured the population-weighted centroids (Luo and Wang, 2003) for Utah ZIP Codes using the 2010 Census Blocks population. Census blocks provide the most granular information about the distribution of the population throughout a geographic area. Population-weighted centroids allow us to account for the distribution of population within a ZIP Code rather than simply estimating the geometric center of ZIP Codes. (Luo and Wang, 2003) We used the population of all census blocks which fall within a ZIP Code boundary as weights to calculate the centroid for that ZIP Code. For supply, we needed the location of food providers, including 121 Food Pantries, 78 Congregate Meals/Nutrition Sites, 36 Home Delivered Meals, 15 Food Production Support Services, and 14 Soup Kitchens. We geocoded those addresses using ArcGIS Pro. Each food provider represents one unit of supply.

Finally, we adopted the 2-step floating catchment area (2SFCA) method (Luo and Wang, 2003) to measure accessibility at the ZIP Code level. 2SFCA method addresses the limitations of previous methods such as the population-to-provider ratio which does not consider travel impedance, or measuring distance to the nearest provider which assumes that residents would always use the nearest provider. 2SFCA has two steps: in the first step, we created a catchment area (i.e., 10-minute drive time using the road network and speed limit data (UGRC, 2022) and network analysis available in ArcGIS Pro 2.7.3), then calculated a supply/demand ratio based on the population size within the catchment. In the second and final step, we created a catchment area with the same travel time used in step one for each population-weighted centroid, searched for the food providers within that catchment area, and summed their supply/demand ratio to represent accessibility to food providers at the ZIP Code level.

## 3 Results

Between January 2019 and April 2020, 2,472 adult patients were screened for social needs. After removing missing data (either because patients did not finish the 10 questions or there were missing data in the demographics information), 2,290 records were included in our analyses. These dropped records were not significantly different from our final sample. Descriptive statistics of our data are provided in Table 2.

**Table 2:** Baseline characteristics of adult patients screened for social needs (n = 2,290) and those with an unmet food need (n = 495) in an academic health sciences center ED in Utah.

**Variable**	**Total n (%) for categorical variables and mean^&^ (standard deviation) for numerical variables**	**Characteristics of patients with unmet food needs: Total n (%) for categorical variables and mean^&^ (standard deviation) for numerical variables**	P-Value from a two-sample *t*-test* or a chi-squared test
Unmet Food Needs (categorical)	495 (21.61)	N/A	N/A
Age (numerical)	^&^ 44.66 (17.76)	^&^ 41.95 (13.95)	< 0.001*
Female (categorical)	1263 (55.15)	243 (49.09)	**0.0025**
Hispanic (categorical)	329 (14.36)	96 (19.39)	**< 0.001**
Non-White (categorical)	473 (20.65)	127 (25.65)	**0.0023**
ED Utilization (numerical)	^&^ 1.22 (1.66)	^&^ 1.45 (2.06)	< 0.001*
PCP Utilization (numerical)	^&^ 0.53 (1.56)	^&^ 0.43 (1.66)	< 0.001*
Hospital Utilization (numerical)	^&^ 0.15 (0.50)	^&^ 0.14 (0.56)	< 0.001*
Nonmetro ZIP Codes (categorical)	165 (7.2)	38 (7.67)	0.71
Accessibility to Food Providers for a 10-minute drive time (numerical)	^&^ 0.0001 (0.00011)	^&^ 0.00012 (0.00014)	< 0.001*
SNAP Utilization Rate (numerical)	^&^ 0.074 (0.047)	^&^ 0.092 (0.048)	< 0.001*
Transportation Needs (categorical)	252 (11)	160 (32.32)	**< 0.001**
Medical Visit Cost Needs (categorical)	488 (21.31)	273 (55.15)	**< 0.001**
Medication Needs (categorical)	404 (17.64)	247 (49.89)	**< 0.001**
Utilities Needs (categorical)	552 (24.1)	344 (69.49)	**< 0.001**
Housing Needs (categorical)	348 (15.19)	243 (49.09)	**< 0.001**
Employment Needs (categorical)	431 (18.82)	242 (48.88)	**< 0.001**
Childcare/Eldercare Needs (categorical)	153 (6.68)	97 (19.59)	**< 0.001**

P-values are reported for univariate analysis between patients who reported unmet food needs vs those who did not by using a *t*-test for numerical variables and a chi-square test for categorical variables. Boldface indicates statistical significance (P < 0.05).

For ZIP Code-level accessibility to food providers, the 0.0001 ratio indicates that our ED patients are living in ZIP Codes that on average provide access to 0.0001 food providers within a 10-minute drive time (translates to 1 food provider per 10,000 people).

For SNAP utilization, the 0.074 ratio indicates that our ED patients are living in ZIP Codes that on average %7.4 of their households have used SNAP benefits in the last year.

**Fig. 1:**
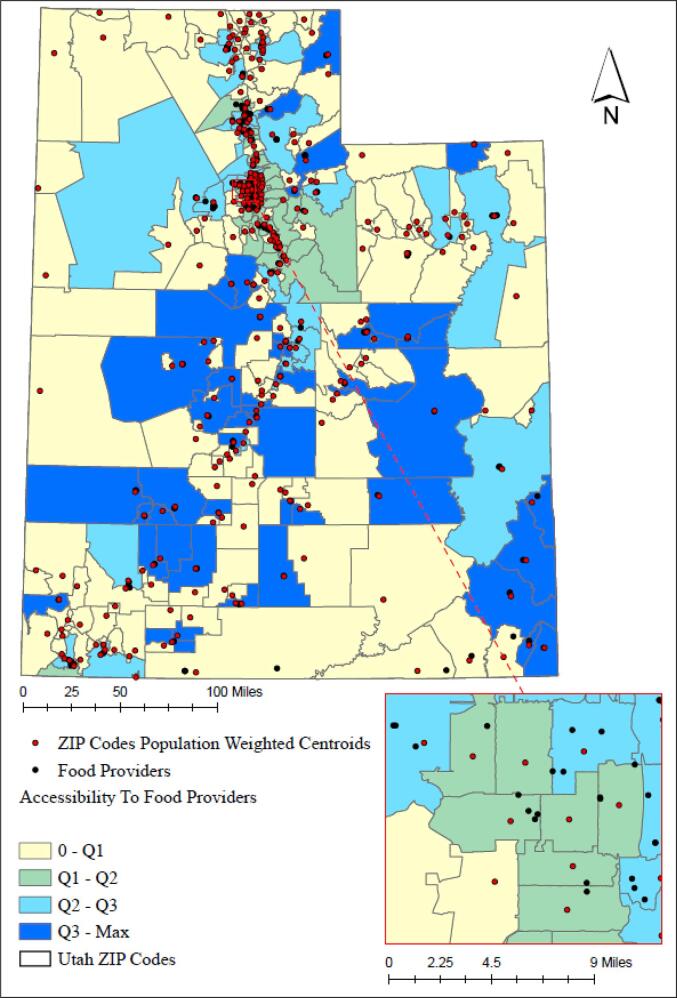
Accessibility to food providers at the ZIP Code level for a 10-minute drive time from ZIP Codes’ population-weighted centroids. Classification method: quartile – (yellow = worst access to food providers - Dark blue = best access to food providers). The first quartile is 0; second quartile is 0.000059, third quartile is 0.00034, and the max is 0.0052 (translates to 52 food providers per 10,000 people for a 10-minute drive time). The zoomed section is Salt Lake City. (For interpretation of the references to colour in this figure legend, the reader is referred to the web version of this article.)

Fig. 1 illustrates the GIS analysis results, which illustrate the 10-minute drive time spatial accessibility to food providers at the ZIP Code level. The higher an accessibility score is for a ZIP Code, the better spatial accessibility the residents of that ZIP Code have to food providers. The accessibility score is shown using the quartile method. The first quartile is equal to zero, therefore, the yellow areas on the map highlight ZIP Codes with zero access to food providers within a 10-minute drive time from their population-weighted centroids. Also on average, Utah ZIP Codes provide access to 2.7 food providers per 10,000 people.

We found that 10 % of the total variation in the outcome variable (i.e., unmet food needs) could be accounted for by the ZIP Codes alone, as reported by the intraclass correlation coefficient (ICC) (Nakagawa et al., 2017) in Model 1 (see Table 3). Also introducing the random intercept improved our model, reducing the Akaike Information Criterion (AIC) by 74. Including individual-level demographic factors reduced the ICC by 2 % (see Model 2). Including the ZIP Code-level population characteristics to Model 2 decreased the ICC to 3 % (see Model 3). Finally, including the other social needs into our model resulted in an ICC of zero (see Model 4). In Models 3 and 4, we also investigated the improvement of our models by introducing random slopes for SDoH, however, neither the ICC nor the AIC improved. Model 4 in Table 3 illustrates the importance and impact of other social needs on unmet food needs irrespective of demographic information. Besides social needs, only PCP utilization and SNAP participation were significantly associated with unmet food needs.

**Table 3:** Multilevel logistic regression with covariates that were associated with unmet food needs.

	**Model 0 (fixed intercept, no slope)**	**Model 1 (random intercept, no slope)**	**Model 2 (Model 1 + demographic factors)**	**Model 3 (Model 2 + Population characteristics)**	**Model 4 (Model 3 + Social Needs)**
Variables	AIC = 2392	AIC = 2318 ICC = 10 %	ICC = 8 %	ICC = 3 %	ICC = 0 %
Intercept (only the fixed effect)	**0.27 (0.24**–0.3)**	**0.23 (0.19**–0.28)**	**0.34 (0.19**–0.6)**	**0.18 (0.1**–0.33)**	**0.03 (0.01**–0.07)**
Age			0.46 (0.21–1.02)	**0.44 (0.2**–0.97)*	0.75 (0.25–2.27)
Female (ref: male)			**0.75 (0.61**–0.93)**	**0.75 (0.61**–0.92)**	1.08 (0.81–1.43)
Hispanic (ref: non-Hispanic)			1.28 (0.92–1.78)	1.25 (0.9–1.74)	1.08 (0.7–1.67)
Non-White (ref: white)			1.03 (0.77–1.37)	0.98 (0.73–1.3)	1.09 (0.77–1.61)
ED Utilization			**3.47 (1.47**–8.18)**	**3.27 (1.39**–7.7)**	1.98 (0.66–5.89)
PCP Utilization			0.53 (0.28–1.01)	**0.53 (0.28**–1)*	**2.54 (1.17**–5.48)*
Hospital Utilization			0.63 (0.31–1.26)	0.64 (0.31–1.28)	1.02 (0.42–2.49)
Nonmetro ZIP Codes (ref: metro ZIP Codes)				1.22 (0.73–2.02)	1.14 (0.66–1.95)
Accessibility to food providers				2.06 (0.19–22.63)	3.85 (0.26–56.95)
SNAP Utilization				**34.09 (10.31**–112.73)**	**3.39 (1.06**–10.78)*
Transportation Needs					**1.71 (1.15**–2.55)**
Medical Visit Cost Needs					**1.56 (1.11**–2.22)*
Medication Needs					**3.27 (2.34**–4.56) **
Utilities Needs					**5.98 (4.49**–7.98)**
Housing Needs					**3.97 (2.68**–5.38)**
Employment Needs					**1.76 (1.27**–2.44)**
Childcare/Eldercare Needs					**1.76 (1.09**–2.86)*

Data are reported as adjusted odds ratio (aOR) (95% confidence interval).

Starting from Model 1 with a random intercept and no effect; and stepwise inclusion of demographic factors (Model 2), population characteristics (Model3), and social needs status (Model 4).

The reference level for social needs is the lack of that need.

Boldface indicates statistical significance at *P < 0.05, and **P < 0.01.

## 4 Discussion

Our research was informed by a lack of knowledge about factors associated with food insecurity from a social needs perspective. Lack of screening for food insecurity in U.S. healthcare settings (De Marchis et al., 2019); (Fraze et al., 2019) and lack of clinicians’ and patients’ time during clinical visits (De Marchis et al., 2019); (Pooler et al., 2018) indicate the need and importance of promptly identifying the most important factors associated with food insecurity. Asking about patients’ social needs and identifying associated needs with food insecurity will allow us to tailor interventions for our population. Using the SINCERE tool takes <80 s, (Guo et al., 2021, Wallace et al., 2020) hence, these 10 questions will not create too much burden. In addition, the questions have been simplified based on clear communication and low-literacy principles and the tool could be administered with low to zero assistance from ED staff. (Wallace et al., 2020).

Considering that 10 % of the total variation in the outcome variable (i.e., unmet food needs) could be accounted for by ZIP Codes alone, we adopted a multilevel logistic regression model with a random intercept (Model 1). Then, in a stepwise approach, we explored the improvement of our model by adding the fixed effect of individual-level demographic information and health utilization (Model 2), population-level SDoH (Model 3), and social needs (Model 4) to Model 1. In Models 3 and 4, we investigated the improvement of our model by introducing random slopes for ZIP Code-level SDoH, however, neither the ICC nor the AIC improved. This observation indicates that random slopes are not required and a model with random intercept and fixed slopes accounts for variation in the outcome variable at the ZIP code level. Model 3 highlights the importance of age and sex in determining food insecurity, as older adults (aOR 0.44) and females (aOR 0.75) had lower odds of reporting unmet food needs. However, none of these factors remained significant after introducing other social needs to our model (see Model 4 in Table 3). Also, the ICC was 0 % after including the social needs, suggesting that the social needs fixed effects account for most of the variation within- and between- ZIP Codes. The significance of ZIP Code-level SNAP utilization could indicate that the SNAP’s support might not be enough to fully address unmet food needs, or our sample population was not representative of the ZIP Code-level SNAP utilization; however, considering its wide confidence interval, the estimate is relatively unstable. Moreover, accessibility to food providers was not identified as significant in any models. While the availability of food pantries can increase access to healthy food options, (Mabli et al., 2013) availability and spatial accessibility are not the only factors affecting access as food pantries could have limited open hours and provide limited and low-quality foods. (Ginsburg et al., 2019) Food pantries rely on donations, hence lack of donation could deteriorate the impact of spatial accessibility. Also, lack of accommodation (welcoming environment, respecting the dignity of clients and their preferences) can decrease access to food. (Ginsburg et al., 2019, Sanderson et al., 2020) These factors are hidden from our research team, which might have yielded the insignificant impact of accessibility to food providers on the outcome variable.

Our final results (Model 4) identified the most important associated factors with food insecurity as utilities (aOR 5.98), housing (aOR 3.97), and medication needs (aOR 3.27) based on the precision (i.e., narrow confidence interval) and higher impact (i.e., aOR). Accordingly, food insecurity did not seem to be independently correlated with race/ethnicity and other demographic factors. In another word, while individual-level demographics and health utilization factors and ZIP-Code level SDoH could lead us to the identification of patients at risk of food insecurity, social needs, specifically utilities, housing, and medication are more strongly associated with food insecurity. One could think that lack of income is the common reason for social needs but unemployment or childcare/eldercare needs are not related to lack of financial resources. It is important to screen these needs so that we could tailor our interventions based on the patients’ needs.

In addition, healthcare utilization has been used as a risk factor for food insecurity; for instance, high ED visits and hospitalization had been linked to food insecurity. (Peltz and Garg, 2019, Berkowitz et al., 2018) However, our results indicate that healthcare utilization is not a precise indicator of food insecurity; patients with reported food insecurity were not tied to ED use or hospitalization (See Model 4). On the other hand, PCP users tend to report higher unmet food needs, maybe because PCP has less of a cost barrier compared to ED and hospitals. The significance of PCP utilization in Model 4 highlights the providers’ opportunity in intervening and addressing food insecurity during a PCP visit. PCP can provide some resources in the office or discuss the needs with patients in order to connect them to resources. Primary care settings have been also identified as an appropriate and ideal setting to screen for food insecurity compared to other clinical settings. (De Marchis et al., 2019) However, we should address PCP’s lack of knowledge about available resources and consider food insecurity as a top public health priority to encourage insurance companies to reimburse PCP for screening for food insecurity. (Pooler et al., 2018, de la Vega et al., 2019, Cole et al., 2022, Taher et al., 2021) Also notice that the impact of PCP visits on reporting unmet food needs changed from aOR 0.53 in Model 2 and 3 to aOR 2.54 in Model 4. This huge difference highlights the importance of considering other social needs in our food insecurity screening.

### 4.1 Limitations and future research

Due to time restrictions during ED visits and based on several meetings with different stakeholders, only one question was used for each social need. While our models identified significant associations between covariates and an unmet food need, the causal relationship between them is unknown. In addition, in accessibility studies, researchers use different measures to distinguish the amount of service each supplier provides but, in our study, we did not have any indicator to differentiate the supply strength of food providers. Moreover, there are no published data regarding how far people are willing to travel to get to food providers, hence future research could explore and conduct sensitivity analyses around travel time. Our population sample is representative of the Utah population, hence future research could replicate our analyses in other states. Further research is also required to identify the optimal setting and workforce to screen for food insecurity.

## 5 Conclusion

Understanding and addressing individual-level food insecurity requires investigation of factors at multi-levels, specifically, individual-level social needs. While clinicians are interested in shorter screeners due to time restrictions, there is a tradeoff between time and precision. As the results suggest, we cannot address food insecurity by only addressing unmet food needs. Other social needs associated with food insecurity create a closed loop of problems hard to address. Having multiple social needs, irrespective of patient demographics and population-level SDoH, impact patients’ food insecurity significantly. In addition, our results suggest that primary care may be an effective location for addressing food insecurity in health care settings.

## CRediT authorship contribution statement

**Nasser Sharareh:** Conceptualization, Investigation, Project administration, Resources, Methodology, Data curation, Formal analysis, Visualization, Writing – original draft, Validation. **Andrea S. Wallace:** Conceptualization, Data curation, Writing – review & editing, Supervision, Funding acquisition. **Ben J. Brintz:** Methodology, Writing – review & editing, Validation. **Neng Wan:** Methodology, Writing – review & editing, Validation. **Jia-Wen Guo:** Data curation, Validation, Writing – review & editing. **Bob Wong:** Project administration, Data curation, Writing – review & editing, Supervision.

## Declaration of Competing Interest

The authors declare that they have no known competing financial interests or personal relationships that could have appeared to influence the work reported in this paper.

## Data Availability

The data that has been used is confidential.

## References

[b0005] Alderwick H., Gottlieb L.M. (2019). Meanings and misunderstandings: a social determinants of health lexicon for health care systems. Milbank Quarterly.

[b0010] Berkowitz S.A., Seligman H.K., Meigs J.B., Basu S. (2018). Food insecurity, healthcare utilization, and high cost: a longitudinal cohort study. Am. J. Managed Care.

[b0015] Castrucci B., Auerbach J. (2019). Meeting individual social needs falls short of addressing social determinants of health. Health Affairs Blog..

[b0020] Census. Explore Census Data. 2022. Available at: https://data.census.gov/cedsci/, Accessed 2022.

[b0025] Chilton M., Black M.M., Berkowitz C. (2009). Food insecurity and risk of poor health among US-born children of immigrants. Am. J. Public Health.

[b0030] Cole M.B., Nguyen K.H., Byhoff E., Murray G.F. (2022). Screening for social risk at federally qualified health centers: a national study. Am. J. Prev. Med..

[b0035] Coleman-Jensen A, Rabbitt MP, Gregory CA, Singh A. Household Food Security in the United States in 2020. 202Available at: https://www.ers.usda.gov/webdocs/publications/102076/err-298.pdf?v=9888.4.

[b0040] de la Vega P.B., Losi S., Martinez L.S. (2019). Implementing an EHR-based screening and referral system to address social determinants of health in primary care. Med. Care.

[b0045] De Marchis E.H., Torres J.M., Fichtenberg C., Gottlieb L.M. (2019). Identifying food insecurity in health care settings: a systematic scoping review of the evidence. Family Commun. Health.

[b0050] Escobar E.R., Pathak S., Blanchard C.M. (2021). Peer reviewed: screening and referral care delivery services and unmet health-related social needs: a systematic review. Prevent. Chronic Dis..

[b0055] Ettinger de Cuba S., Chilton M., Bovell-Ammon A. (2019). Loss of SNAP is associated with food insecurity and poor health in working families with young children. Health Aff..

[b0060] Feeding America. *Hunger in America 2014.* 2014. Available at: https://www.feedingamerica.org/sites/default/files/2020-02/hunger-in-america-2014-full-report.pdf, Accessed 2014.

[b0065] Feeding America. Map the Meal Gap. 2020. Available at: https://www.feedingamerica.org/sites/default/files/2020-06/Map%20the%20Meal%20Gap%202020%20Combined%20Modules.pdf, Accessed 2020.

[b0070] Fraze T.K., Brewster A.L., Lewis V.A., Beidler L.B., Murray G.F., Colla C.H. (2019). Prevalence of screening for food insecurity, housing instability, utility needs, transportation needs, and interpersonal violence by US physician practices and hospitals. JAMA Network Open..

[b0075] Gelman A., Hill J. (2006).

[b0080] Ginsburg Z.A., Bryan A.D., Rubinstein E.B. (2019). Unreliable and difficult-to-access food for those in need: A qualitative and quantitative study of urban food pantries. J. Community Health.

[b0085] Glasheen S. *Meeting Social Needs and Addressing Social Determinants of Health.* 2019. Available at: https://www.uhccommunityandstate.com/content/blog-post/sarah-glasheen-posts/meeting-social-needs-and-addressing-social-determinants-of-health#:∼:text=While%20the%20concepts%20of%20%E2%80%9Csocial,across%20the%20health%20care%20community.&text=Social%20needs%20focus%20on%20the,well%2Dbeing%2C%20and%20safety., Accessed 2019.

[b0090] Gottlieb L.M., Hessler D., Long D. (2016). Effects of social needs screening and in-person service navigation on child health: a randomized clinical trial. JAMA Pediatrics.

[b0095] Green K., Zook M. (2019). When talking about social determinants, precision matters. Health Affairs Blog..

[b0100] Guo J.-W., Wallace A.S., Luther B.L., Wong B. (2021). Psychometric evaluation of the screener for intensifying community referrals for health. Eval. Health Prof..

[b0105] Hager E.R., Quigg A.M., Black M.M. (2010). Development and validity of a 2-item screen to identify families at risk for food insecurity. Pediatrics.

[b0110] Heller C.G., Rehm C.D., Parsons A.H., Chambers E.C., Hollingsworth N.H., Fiori K.P. (2021). The association between social needs and chronic conditions in a large, urban primary care population. Prev. Med..

[b0115] Henrikson N.B., Blasi P.R., Dorsey C.N. (2019). Psychometric and pragmatic properties of social risk screening tools: a systematic review. Am. J. Prev. Med..

[b0120] Hsu C., Cruz S., Placzek H. (2020). Patient perspectives on addressing social needs in primary care using a screening and resource referral intervention. J. Gen. Intern. Med..

[b0125] Luo W., Wang F. (2003). Measures of spatial accessibility to health care in a GIS environment: synthesis and a case study in the Chicago region. Environ. Plann. B: Plann. Design.

[b0130] Mabli J., Jones D., Kaufman P. (2013). Characterizing food access in America: Considering the role of emergency food pantries in areas without supermarkets. J. Hunger Environ. Nutrit..

[b0135] Martinez S.M., Webb K., Frongillo E.A., Ritchie L.D. (2018). Food insecurity in California’s public university system: What are the risk factors?. J. Hunger Environ. Nutrit..

[b0140] Morland K., Wing S., Roux A.D., Poole C. (2002). Neighborhood characteristics associated with the location of food stores and food service places. Am. J. Prev. Med..

[b0145] Morris, L.M., Smith, S., Davis, J., Null, D.B., 2016. The prevalence of food security and insecurity among Illinois university students. *J. Nutrit. Educ. Behav.* 48(6):376-382. e371. 10.1016/j.jneb.2016.03.013 27118138

[b0150] Nakagawa S., Johnson P.C., Schielzeth H. (2017). The coefficient of determination R 2 and intra-class correlation coefficient from generalized linear mixed-effects models revisited and expanded. J. R. Soc. Interface.

[b0155] Peltz A., Garg A. (2019). Food insecurity and health care use. Pediatrics.

[b0160] Pooler J.A., Hoffman V.A., Karva F.J. (2018). Primary care providers’ perspectives on screening older adult patients for food insecurity. J. Aging Soc. Policy.

[b0165] Rogers A.J., Hamity C., Sharp A.L., Jackson A.H., Schickedanz A.B. (2020). Patients’ attitudes and perceptions regarding social needs screening and navigation: multi-site survey in a large integrated health system. J. Gen. Intern. Med..

[b0170] Sanderson J., Martin K.S., Colantonio A.G., Wu R. (2020). An outcome evaluation of food pantries implementing the More than Food framework. J. Hunger Environ. Nutrit..

[b0175] Schanzenbach, D.W., Bauer, L., Nantz, G., 2016. *Twelve facts about food insecurity and SNAP.* Brookings Institution Washington DC.

[b0180] Sharareh, N., Wallace, A.S., 2022. Applying a Health Access Framework to Understand and Address Food Insecurity. Paper presented at: Healthcare. 10.3390/healthcare10020380 PMC8872536 35206993

[b0185] Smith D., Thompson C., Harland K., Parker S., Shelton N. (2018). Identifying populations and areas at greatest risk of household food insecurity in England. Appl. Geogr..

[b0190] Taher S., Persell S.D., Kandula N.R. (2021). Six recommendations for accelerating uptake of national food security screening in primary care settings. J. Gen. Intern. Med..

[b0195] Tong S.T., Liaw W.R., Kashiri P.L. (2018). Clinician experiences with screening for social needs in primary care. J. Am. Board Family Med..

[b0200] UGRC. Streen Network Analysis. 2022. Available at: https://gis.utah.gov/data/transportation/street-network-analysis/, Accessed 2022.

[b0205] United Way. United Way of Salt Lake. 2022. Available at: https://uw.org/, Accessed 2022.

[b0210] USDA's Economic Research Service (ESR). Rural-Urban Commuting Area Codes. 2020. Available at: https://www.ers.usda.gov/data-products/rural-urban-commuting-area-codes/, Accessed 2022.

[b0215] Wallace A.S., Luther B., Guo J.-W., Wang C.-Y., Sisler S., Wong B. (2020). Implementing a social determinants screening and referral infrastructure during routine emergency department visits, Utah, 2017–2018. Prevent. Chronic Dis..

[b0220] Yousefi-Rizi L., Baek J.-D., Blumenfeld N., Stoskopf C. (2021). Impact of housing instability and social risk factors on food insecurity among vulnerable residents in San Diego County. J. Community Health.

