# Supplementary material for: Associated factors with patient-reported unmet food needs among emergency department adult patients – A social need perspective
Source: Prev Med Rep. 2022 Sep 7;29:101974. doi: 10.1016/j.pmedr.2022.101974 (PMC9502286; doi:10.1016/j.pmedr.2022.101974)
Supplement: Supplementary data 1 [file mmc1.docx]

**Title: Associated factors with patient-reported unmet food needs among emergency department adult patients – a social need perspective**

# Appendix

## Social needs

All the social needs are binary variables. For each social need, patients were asked one question and if the answer to that question was Yes, then they were identified with that social need. For instance, for medication, a patient was asked “Did you not take medications to save money?” and if he/she said Yes, then it means that he/she had a Medication need.

In the models, the reference variable for each of those 10 social needs is no needs, indicating that patients answered No to the corresponding questions.

## Individual-Level Demographics

Age: continuous variable

Gender: binary variable (male as reference variable)

Race: binary variable (white as reference variable)

Ethnicity: binary variable (non-Hispanic as reference variable)

## Individual-Level Healthcare Utilization in the Past 90 Days

ED: continuous variable

PCP: continuous variable

Hospitalization: continuous variable

## ZIP Code Level Data

Accessibility to food providers: rates ranging from 0 to 1 and estimated by a geographic information systems method defined in the main article

Patient’s ZIP Code’s metro/nonmetro status: binary variable (metropolitan as reference variable) defined by the rural-urban commuting area codes

Median household income (Census Table S1903): continuous variable indicating the median household income

Education level (Census Table S1501): population ages 25+ with high school or higher education divided by total population ages 25+ of ZIP Code

SNAP utilization (Census Table S2201): number of households receiving SNAP in the last year in each ZIP Code divided by all the households within each ZIP Code

Health insurance (Census Table S2701): the number of uninsured divided by the total population of ZIP Code
